# Prolongation of mitosis is associated with enhanced endogenous DNA damage in fission yeast.

**DOI:** 10.17912/micropub.biology.000911

**Published:** 2023-07-13

**Authors:** Ishutesh Jain, Phong T. Tran

**Affiliations:** 1 Institut Curie, PSL Université, Sorbonne Université, CNRS UMR 144, Paris 75005, France; 2 Simons Centre for the Study of Living Machines, National Centre for Biological Sciences - TIFR, Bangalore 560065, India; 3 University of Pennsylvania, Department of Cell and Developmental Biology, Philadelphia, PA 19104, USA

## Abstract

Mitosis is usually shorter than other phases of the cell cycle and maintains a consistent duration despite variations in cell size and spindle size. This suggests the existence of a compensatory mechanism that ensures a short duration, possibly as a protective measure against irreversible damage, such as DNA damage. To explore the link between prolonged mitosis and DNA damage, we develop a microscopy-based assay utilizing Rad52-GFP as a marker for mitotic DNA damage. Through this assay, we provide evidence that mutants with prolonged mitosis exhibit increased Rad52 puncta, indicating an elevation in endogenous DNA damage.

**Figure 1. A method for visualizing mitotic DNA damage reveals a positive correlation between prolonged mitosis duration and increased DNA damage.**
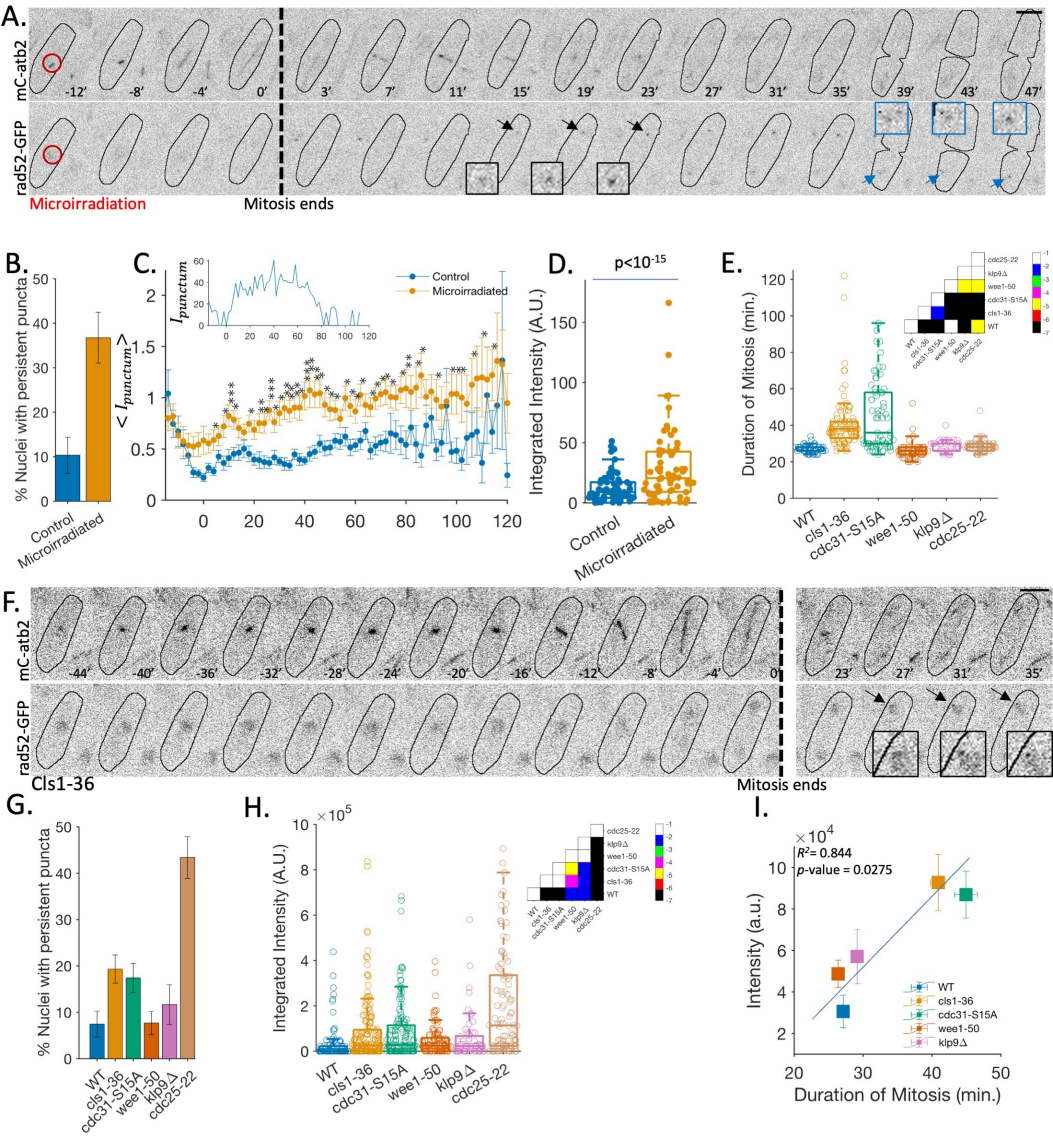
A. Time-lapse images of a wild-type cell, expressing mCherry-Atb2 (tubulin) and Rad52-GFP (DNA damage marker), that was micro-irradiated with an ultraviolet laser during mitosis. The red circle indicates the irradiation location, the arrow points to the punctum location, and the square insets are zoom-in of the punctum. The dotted line marks the end of the mitosis (top). In mitotic cells, Rad52-GFP punctum appears after the completion of mitosis in one (or both) daughter nuclei. In contrast, in the interphase cells, the Rad52 punctum appears within minutes after micro-irradiation. Scale bar = 5 µm. B. Quantification of the occurrence of persistent Rad52-GFP punctum post-mitosis in control and micro-irradiated nuclei. Error bars represent Standard Error. C. (inset) Example of a time series of punctum intensity (I _punctum_ ) in a nucleus micro-irradiated during mitosis, measured using a semi-automated spot tracking program (see methods). Zero marks the time when mitosis completes. (main) Mean relative punctum intensity (〈I _punctum (t)_ 〉= 1/n ∑ _n_ [(I _punctum(t)_^i^ )/(I _nucleus(t)_^i^ )] -1 ) over time in control and micro-irradiated cells. The irradiated nuclei show significant differences in mean intensity between 15 to 90 min. after mitosis ended. Error bars represent Standard Error, and *** for p≤10 ^-4^ , ** for p≤10 ^-3^ , and * for p≤0.05, based on the Wilcoxon rank-sum test. D. DNA damage observed in a single nucleus is estimated using the integration of punctum intensity for 90 min from the end of the mitosis. Statistics is estimated using the Wilcoxon rank-sum test. E. Quantification of the duration of mitosis in different strains, measured from the disappearance of interphase MT to the breakdown of the mitotic spindle in anaphase. (Inset) p-values obtained using the rank-sum test across strains. The color bar indicates the scale of p-values, represented as the power of 10. F. Examples of post-mitotic Rad52-GFP punctum observations in strains with longer mitosis duration. G. Occurrence of persistent Rad52-GFP punctum in the daughter nuclei after the end of mitosis in different strains identified through visual inspection. The error bars represent the Standard Error. H. Estimation of DNA damage observed in a single nucleus, based on the integration of punctum intensity over 90 minutes. (Inset) p-values obtained using the rank-sum test across strains. The color bar indicates the scale of p-values, represented as the power of 10. I. Correlation between mitotic duration and DNA damage. Regression analysis coefficient of determination R ^2^ and p-value are shown. The error bars represent the Standard Error.

## Description

Mitosis is a tightly regulated and precise process that ensures the accurate division of replicated genomes into daughter cells. Errors in chromosome segregation during mitosis can have serious consequences, such as aneuploidy. Despite these risks, mitosis is relatively short compared to other phases of the cell cycle, typically lasting around 30 minutes (Araujo et al., 2016) . Interestingly, research has shown a correlation between spindle size and cell size in various systems (Farhadifar et al., 2015; Good et al., 2013; Hara and Kimura, 2009; Krüger et al., 2019). As the spindle size scales with the cell, the rate of spindle assembly and elongation increases to maintain a constant duration of mitosis (Farhadifar et al., 2015; Hara and Kimura, 2009; Krüger et al., 2019). This implies the presence of a compensatory mechanism that contributes to the short duration of mitosis. These observations suggest that the short duration of mitosis serves as a protective mechanism against potential irreversible damage, such as DNA damage. To investigate this hypothesis, we employ fission yeast as a model system, aiming to explore the relationship between prolonged mitosis and DNA damage.

The DNA repair response is suppressed during mitosis, resulting in a lack of methods for rapidly assessing DNA damage specifically associated with this phase (Hustedt and Durocher, 2017) . We hypothesize that the DNA damage occurring during mitosis is likely to be detected and repaired after mitosis ends. We first implemented a method to visualize and quantify mitotic DNA damage. To identify factors that can quantify mitotic DNA damage, we conducted a micro-screen for repair factors using the following approach: we micro-irradiated a specific point within the nucleus during early mitosis (identified by a small spindle and visualized with the tubulin mCherry-Atb2), using an ultraviolet (UV) laser. We then monitored for the appearance of fluorescent punctum during and after mitosis ( Fig. 1A ). Through this strategy, we identified Rad52-GFP as a reporter of mitotic DNA damage. Rad52 facilitates the exchange of the single-strand DNA binding protein RPA with the recombinase Rad51 during homologous recombination (HR)-mediated DNA repair (Hustedt and Durocher, 2017; Mortensen et al., 2009) . In interphase, Rad52 punctum formed within minutes of UV micro-irradiation. However, in micro-irradiated mitotic nuclei, Rad52 punctum appeared after mitosis ended ( Fig. 1A ). Only 2 out of 50 micro-irradiated mitotic nuclei showed Rad52 punctum during mitosis. The manifestation of Rad52 punctum after mitosis varied from a few minutes to up to 90 minutes. This likely reflects the gradual activation of HR-mediated DNA repair after the brief G1 phase, which requires Rad52 activity (Karanam et al., 2012) . Typically, only one of the daughter nuclei showed punctum (42 out of 50), suggesting localized DNA damage due to micro-irradiation. Rarely did we observe more than one punctum in the daughter nuclei, corroborating this. We observed Rad52 punctum in around 10% of control nuclei after mitosis, which could result from a combination of basal endogenous DNA damage and stalled replication forks ( Fig. 1B ) (Haber, 2013; Tubbs and Nussenzweig, 2017) . However, around 40% of daughter nuclei from micro-irradiated mitotic cells exhibited persistent Rad52 punctum ( Fig. 1D ), indicating a response to DNA damage during mitosis and suggesting post-mitotic Rad52 punctum as a marker for mitotic DNA damage. Using a semi-automated segmentation pipeline, we segmented cells, identified punctum (see Methods), and examined the dynamics of Rad52 punctum. We observed consistent intensity of Rad52 punctum throughout their persistence, indicating limited photobleaching in our experiments ( Fig. 1C inset ). The difference in mean punctum intensities between micro-irradiated and control cells peaked between 20-50 minutes after mitosis ended ( Fig. 1C ). Approximately 100 minutes after mitosis termination, the difference between the two vanished, suggesting that most repair punctum formed within this period after mitosis. Additionally, integrating the intensity of identified punctum over 90 minutes after mitosis revealed significant differences between micro-irradiated and control cells ( Fig. 1D ). This integrated intensity can serve as an objective measure to quantify DNA damage associated with mitosis.

Next, we performed live cell microscopy on several mutants in fission yeast known for their prolonged mitosis, using a background of Rad52-GFP mCherry-Atb2 (referred to as Wildtype, WT). As controls, we included mutants with spindle scaling properties that exhibited similar mitotic durations to WT. The mutants were as follows:

1. *cls1-36* : a mutation in the microtubule (MT) polymerase CLASP, resulting in extended metaphase (Bratman and Chang, 2007) .

2. *cdc31-S15A* : a mutation affecting the spindle pole body scaffolding protein, leading to slow-resolving monopolar spindles (Bouhlel et al., 2015) .

3. *klp9∆* : deletion of the anaphase-specific kinesin-6 motor involved in spindle elongation (Krüger et al., 2019).

4. *cdc25-22* : a cell cycle mutant with delayed G2-M transition, resulting in longer cells.

5. *wee1-50* : a mutant that shortens the cell cycle by inducing early G2-M transition, leading to shorter cells (Russell and Nurse, 1986) .

The *cls1-36* and *cdc31-S15A* mutants exhibited significantly longer prophase durations compared to WT (approximately twice as long, around 42 minutes, Fig. 1E ). The *klp9∆* mutant showed a longer anaphase, while the cell cycle mutants, *cdc25-22* and *wee1-50* , had different cell sizes but exhibited mitosis durations similar to WT cells (27 minutes, Fig. 1E ).

In mutants with prolonged mitosis ( *cls1-36* and *cdc31-S15A* ), many daughter nuclei displayed Rad52-GFP puncta ( Fig. 1F ), similar to the micro-irradiated cells. Visual analysis revealed that approximately 20% of daughter nuclei in the *cls1-36* and *cdc31-S15A* strains exhibited Rad52 puncta, while the WT strain showed puncta in around 8% of daughter nuclei ( Fig. 1G ). Quantification of the integrated intensity of computationally detected puncta confirmed high values of integrated intensity in *cls1-36* and *cdc31-S15A* cells ( Fig. 1H ), further supporting the findings. However, we also observed a large number of Rad52 foci in the large cells of *cdc25-22* mutants. This could be linked to the DNA damage associated with an increase in cell size (Neurohr et al., 2019; Neurohr and Amon, 2020). To avoid potential confounding effects, we excluded the large cell phenotype from our analysis. In the other strains with mitotic durations similar to wild-type cells ( *wee1-50* and *klp9Δ* ), the appearance of Rad52 puncta remained similar to wild-type levels. Taken together, these results demonstrated a positive correlation between mitotic duration and the mean integrated intensity of Rad52 ( Fig. 1I ), suggesting that mutants with increased mitotic duration are more prone to endogenous DNA damage.

Prolonged mitosis in mammalian cell cultures leads to cell cycle arrest, dependent on p53 activation (Uetake and Sluder, 2010) . The specific mechanism of p53 activation in response to prolonged mitosis is unknown (Uetake and Sluder, 2010) . While mitotic errors can trigger p53 activation, DNA damage is recognized as the primary cause (Santaguida and Amon, 2015) . In contrast to mammals, fission yeast does not possess a surveillance mechanism analogous to p53. Therefore, the observation of postmitotic Rad52 puncta in strains with prolonged mitosis indicates the presence of mitosis-specific endogenous DNA damage. Further investigation is needed to understand the underlying mechanisms, which could involve factors such as excessive reactive oxygen species (ROS) or a decline in mitotic cyclin-dependent kinase (M-CDK) activity affecting topoisomerase.

## Methods

Yeast strains and media:

We utilized standard yeast genetics techniques to maintain and perform genetic crosses with the strains used in this study (Hagan et al., 2016) . A complete list of these strains can be found in the reagent table. Typically, cells were cultured on agar plates containing YE5S media at a temperature of 25°C. For microscopy experiments, a small number of cells were inoculated into a liquid YE5S medium the night before the experiment. The culture was then incubated at 25°C with continuous shaking for 16-18 hours until it reached an optical density of approximately 0.5 ± 0.2 absorbance units, at which point the cells were harvested for further analysis.

Microscopy:

For micro-irradiation experiments, imaging was performed using a spinning disk confocal microscope. Specifically, a Nikon Eclipse Ti2 inverted microscope was utilized, equipped with a Nikon CFI Plan Fluor 100x/1.3 NA (UV) objective lens, a Nikon Perfect Focus System (PFS), a Mad City Labs integrated Nano-View XYZ micro- and nano-positioner, a Yokogawa Spinning Disk CSU-X1 unit, a Photometrics Prime BSI sCMOS camera, and ILAS2 Photoablation module (355nm pulsed laser) controlled by Molecular Devices MetaMorph 8.0 and iLas2 software. For GFP and mCherry imaging, solid-state lasers of 488 nm (100 mW) and 561 nm (100 mW) were used. The sample was mounted in YE5S media in 2% agar using custom-made PDMS chambers as previously described (Costa et al., 2013) .

A z-stack comprising a total of 8 focal planes spaced 1 µm apart was acquired in the brightfield channel (exposure time: 20 ms), as well as in the GFP (exposure time: 200 ms) and mCherry (exposure time: 200 ms) channels. The stack was acquired over a period of approximately 3 hours with a time interval of 2 minutes. To micro-irradiate the nuclei during mitosis, the acquisition was paused after 10 minutes, and nuclei in mitosis (identified using the mCherry channel) were ablated on the fly with a 10 ms UV pulse and a 200 nm diameter spot. We used 5% of UV power. In our experiments, we noticed that lowering the UV power did not yield the desired damage, whereas increasing the UV power led to the sudden death of cells.

Mitotic cell imaging was conducted using a similar spinning disk confocal microscope setup. This setup was devoid of the photoablation module and uses Nikon CFI Plan Fluor 100x/1.4 NA objective lens and a Photometrics EMCCD. For GFP and mCherry imaging, we employed solid-state lasers with wavelengths of 488 nm (100 mW) and 561 nm (100 mW), respectively. The images were acquired in a z-stack consisting of 8 focal planes spaced 1µm apart in the brightfield channel (Exposure time: 20 ms), as well as in the GFP (Exposure time: 100 ms) and mCherry (Exposure time: 100 ms) channels. The entire stack was acquired over a period of approximately 3 hours, with a time interval of 2 minutes between each acquisition.

Cell, Nuclei, and spot segmentation:

We utilized the stack of brightfield images to segment cells by detecting the shift in the intensity of the cell boundary (Lugagne et al., 2018) . For cell tracking throughout the time course, we connected the cell at time point *t + 1* to the cell at time point *t* based on maximum overlap. To algorithmically segment the Rad52-GFP foci, we first generated an image by performing a maximum projection in the GFP channel of the segmented cell images. Next, we applied a threshold to the image at a value of *I = μ + 2.5σ* , where *μ* represents the mean and *σ* represents the standard deviation of the GFP signal within the cell. Subsequently, we eliminated all objects smaller than 4 pixels, considering the remaining objects as puncta. This method of Rad52 puncta detection was relatively stringent, resulting in less than 5% false positives compared to visual inspection.

## Reagents

**Table:** 

**Name**	**Genotype**	**Source**
**TP3839**	h+ Rad52-GFP:KanR mC-Atb2:HygR	This Study
**TP3980**	h+ Cls1-36 Rad52-GFP:KanR mC-Atb2:HygR	This Study
**TP3981**	h? Cdc25-22 Rad52-GFP:KanR mC-Atb2:HygR	This Study
**TP4280**	h+ Cdc31-S15A:NatR Rad52-GFP:KanR mC-Atb2:HygR	This Study
**TP4302**	h? Wee1-50 Rad52-GFP:KanR mC-Atb2:HygR	This Study
**TP4460**	h? Klp9Δ:KanR mCAtb2:HygR Rad52-GFP:KanR	This Study
